# Frequency Survey of Brain Metastases and Its Associated Factors Among Iranian Women with Breast Cancer: A Cross-sectional Study in Tehran City

**DOI:** 10.31661/gmj.v13i.3238

**Published:** 2024-02-25

**Authors:** Elham Sadeghi Moghimi, Zeinab Ghanbari, Seyed Abbas Mirmalek, Kamkar Aeinfar, Seyed Alireza Salimi Tabatabaee, Hamid Zaferani Arani, Amir Ghasemi, Ehsan Jangholi, Zahra Abbasy, Mohammad Rahimi, Fereshteh Derayati

**Affiliations:** ^1^ Department of Community Health Nursing, Shiraz, Iran; ^2^ Department of Nursing, School of Nursing and Midwifery, Isfahan University of Medical Sciences, Isfahan, Iran; ^3^ Department of Surgery, Tehran Medical Sciences, Islamic Azad University, Tehran, Iran; ^4^ Department of Neurosurgery, Shariati Hospital, Tehran University of Medical Sciences, Tehran, Iran; ^5^ Faculty of Medicine, Kashan University of Medical Sciences, Kashan, Iran; ^6^ Department of Surgery, Shiraz University of Medical Sciences, Shiraz, Iran; ^7^ Young Researchers and Elite Club, Tehran Medical Sciences, Islamic Azad University, Tehran, Iran; ^8^ Brain and Spinal Cord Injury Research Center, Neuroscience Institute, Tehran University of Medical Sciences, Tehran, Iran; ^9^ Department of Pediatrics, Tehran University of Medical Sciences, Tehran, Iran; ^10^ Student Research Committee, School of Medicine, Mazandaran University of Medical Sciences, Mazandaran, Iran; ^11^ Department of Stem Cells and Developmental Biology, Roudehen Branch, Islamic Azad University, Roudehen, Iran

**Keywords:** Brain, Breast Cancer, Metastasis, Prognosis, Ki-67, Hormonal Receptor

## Abstract

Background: Brain metastases are serious complication of breast cancer (BC) that poses a critical management challenge. Hence, this study aimed to evaluate clinical findings, the status of hormonal receptors, and their correlation with brain metastasis among patients with BC. Materials and Methods: This cross-sectional study was performed on women with BC that was newly diagnosed with brain metastasis from 2020 to 2023. Also, hormonal receptor status (such as p53, estrogen receptor [ER], progesterone receptor [PR], human epidermal growth factor2 [HER2]), histopathological type of BC, duration of disease, type of treatment, local cerebral invasions, and initial presentations were recorded. A P-value less than 0.05 was considered as statistical significance. Results: Of a total of 302 patients, 49 (16.2%) patients had brain metastasis. The mean age of patients was 45.21±8.3 years, which was significantly lower in patients with metastasis (45.96±11.31 vs. 51.13±12.61 years, P=0.008). There was a significant association between the duration of disease in patients with and without brain metastasis (2.76±1.03 vs. 5.55±3.32 years, P=0.002). Also, the most prevalent histopathological type of BC was invasive ductal carcinoma (IDC). Headache was the most common clinical presentation among patients with brain metastasis. In addition, the most and the least common positive receptors among patients with metastasis were Ki-67 (93.87%) and PR (55.1%), respectively. Compared to patients without metastasis, HER2-positive and P53-positive receptors were markedly associated with brain metastasis (P=0.03 and P=0.021, respectively). However, there was no significant association between treatment methods and metastasis status.Conclusion: Patients with younger age, IDC, and positivity of HER2 and P53 receptors were at an increased risk of developing brain metastases.

## Introduction

Breast cancer (BC) is the most common malignancy and cause of cancer-related death among women with a globally rising incidence due to the aging population, alteration of lifestyle, and delayed childbearing [1][2][3]. Although BC is generally more prevalent in developed countries, the overall burden of disease is disproportionately higher in middle- and low-income countries [4].

Recent advances in diagnostic and treatment modalities have allowed for early detection of cancerous lesions and initiation of therapy to prevent the disease progression into metastatic stages [5]. Nonetheless, the course and prognosis of BC are heterogeneous, and dissemination of malignant cells to distant organs depends on multiple factors such as the type of tumor [5].

In approximately 10-15% of BC patients, brain metastasis occurs as the fourth most common site after the bone, lung, and liver [6]. Despite this relatively low rate, brain metastases from BC are second in line only after lung cancer due to the high frequency of BC [7]. As a result of the extended life expectancy and availability of sensitive neuroimaging techniques, brain metastases are increasingly observed even in the context of controlled systemic disease [8][9]. Also, adjuvant and systemic therapies with low penetrance through the blood-brain barrier might elevate the chance of brain metastases [8][10]. Besides progressive neurological deficits impairing the quality of life, brain metastases represent poor outcomes and shorter survival [11].

Extensive literature has focused on the role of genetic subtypes, and molecular mechanisms that determine the aggressiveness of BC as a prognostic factor in the development of brain metastasis [12].

Epidemiological studies have sought to identify associated markers and receptors to provide a better insight into the course of the disease and to improve surveillance and management guidelines for high-risk patients. In the present study, we aimed to address this issue in a sample population of Iranian women with BC and report the demographic characteristics, clinical findings, and the status of hormonal receptors.

## Patients and Methods

Patients

This retrospective cross-sectional study was performed on women with BC who attended to Boo-Ali, Kasra, Lavasani, and Novin hospitals in Tehran, Iran, from December 2020 to August 2023.

Inclusion and Exclusion Criteria

All the known patients with BC older than 18 years who were newly diagnosed with brain metastatic disease were eligible to enroll in the study. Also, patients with concomitantly suffering from other cancers, metastasis to other organs except for the brain, current chemo-radiotherapy, history of brain surgery and/or brain lesions, and history of stroke were excluded.

Data Collection

Data were collected from their medical records. Also, hormonal receptors and antigen status (i.e., estrogen receptor [ER], human epidermal growth factor2 [HER2], and progesterone receptors [PR], P53 and Ki-67 mutants), type of BC, duration of disease, type of treatment, initial presentations, and brain metastasis status were recorded.

Ethical Consideration

The study was approved by the ethical committee of the Tehran Medical Sciences Branch, Islamic Azad University (approval code:9628). Also, the written informed constant was obtained from all patients.

Statistical Analysis

Data are expressed as mean and standard deviation or frequency and percent. Also, data analysis was performed by using Chi-square and t-test via statistical software SPSS version 21 (IBM, Armonk, NY, USA). A P-value=0.05 was considered as significant level.

## Results

**Table:** Table1. Frequency of Hormone Receptors and Treatment Type Among All Patients with Breast Cancer

**Variables**		**Cerebral metastasis**		**P-value**
Yes (n=49)		No (n=253)		
**Hormonal receptor status, n(%)**				
**ER**	Pos	31 (63.26)	207 (81.81)	0.051
	Neg	18 (36.84)	46 (18.19)	
**PR**	Pos	27 (55.1)	200 (79.05)	0.053
	Neg	22 (44.9)	53 (20.95)	
**HER2**	Pos	36 (73.46)	97 (62.17)	0.03
	Neg	13 (26.54)	156 (37.83)	
**P53**	Pos	42 (85.71)	183 (72.33)	0.021
	Neg	7 (14.29)	70 (27.67)	
**Antigen Ki-67**	Pos	46 (93.87)	242 (95.65)	0.08
	Neg	3 (6.13)	11 (4.35)	
**Type of treatment, n(%)**				
Mastectomy		38 (77.55)	165 (65.21)	0.06
Conservative		11 (22.45)	88 (34.79)	0.08

**Pos:** Positive;**Neg:**Negative; **ER:** Estrogen receptor;**PR:**Progesterone receptor; **HER2:**Human epidermal growth factor2

**Figure-1:**
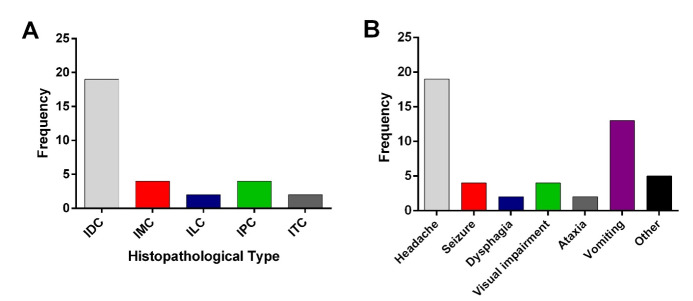
Figure 1. Frequency of histopathological type (A) and initial presentations (B) among BC patients with cerebral metastasis. IDC: Invasive ductal carcinoma; IMC: Invasive medullary carcinoma; ILC: Invasive lobular carcinoma; IPC: Invasive papillary carcinoma; ITC: Invasive tubular carcinoma

The mean age of patients was 45.21±8.3 years (ranged 40-55). In total, 302 patients were enrolled in our study, and among them, 49 (16.2%) patients had brain metastasis. The mean age of the patients with and without brain metastasis was 45.96±11.31 and 51.13±12.61 years, respectively (P=0.008). The mean duration of the disease among all patients was 5.09±23.17 years. Also, there was a significant association between the duration of disease diagnosis among patients with and without metastasis (2.76±1.03 vs. 5.55±3.32 years, P=0.002). As shown in Table-1, the most prevalent type of BC was invasive ductal carcinoma (IDC; 95.34%); invasive lobular carcinoma (ILC) and invasive tubular carcinoma were observed in 4.08% of patients. Also, headache was reported as the most common initial presentation of brain metastasis in 19 (38.77%) patients (Table-1); however, there was no significant association (P˃0.05) Regarding Table-2, in patients with brain metastasis, the most frequent positive receptor was Ki-67 (93.87%); however, PR-positive was diagnosed as the lowest level of hormonal receptors (P˃0.05, Table-2). In contrast, HER2-positive and P53-positive receptors had a significant association with brain metastasis (P=0.03 and P=0.021, respectively). However, other hormonal receptors were more frequent in patients without brain metastasis. Also, there was no significant association between the treatment methods and metastasis status (P˃0.05, Table-2).

## Discussion

In the current research, we studied 302 female patients with BC, and our findings indicate that the incidence of brain metastases in our patients was similar to previous reports [13]. Also, we showed that the mean age of patients with brain metastases was significantly lower than those without brain metastases. Indeed, evidence demonstrated that younger age has been associated with more malignant behaviors of primary BC and a higher chance of brain metastasis [14][15][16]. In line with previous studies, non-specific manifestations, namely headache followed by vomiting, were the most common symptoms [17].

Brain metastases are typically diagnosed based on imaging findings in patients who develop neurological impairment and are managed using symptomatic treatment, surgical excision, and radiotherapy, which add to the overall morbidity [18]. Hence, clinicians must be aware to screen their patients for such complaints and educate them to not underestimate in case they experience related symptoms and possibly associate it with the adverse effects of treatment [19]. Accordingly, imaging strategies should be utilized to monitor the spatial and temporal distribution of dormant cancer cells, metastatic proliferation, and tumor permeability to investigate the true burden of brain metastases in BC, as standard clinical approaches often fail [20].

Our findings indicated that IDC was the most common phenotype BC among patients with brain metastasis. Regarding Tham et al. [21] study-a large survey on patients with metastatic BC- IDC was the primary BC phenotype associated with central nervous system (CNS) metastases. Li et al. [22] showed that ILC was associated with a shorter progression-free survival and, consequently, a worse prognosis; however, in our study only two patients had ILC.Regarding the biological profile, our result indicated that HER2 was significantly higher in the brain metastases group. Currently, the HER2-positive receptor is widely considered as a contributing factor for brain metastases [17][23][24][25][26][27][28][29].

For instance, in a recent meta-analysis study, the prevalence of brain metastasis among patients with HER2-positive BC was estimated up to 24.9% [23]. Although the introduction of trastuzumab, an anti-HER-2 monoclonal antibody, has facilitated extra-cranial control and amplified overall survival, its large molecular weight has limited its ability to cross the blood-brain barrier. Thus, radiotherapy and novel small-molecule radiosensitizing agents targeting HER2 are currently applied for the treatment of brain metastases [30][31].

In addition, our study showed that p53 receptors were markedly more positive in patients with brain metastasis. Previous studies [32][33] revealed the role of p53 in local recurrence and distant metastases, particularly to the CNS. Also, Tazhibi et al. [34] investigated the prognostic factors of distant metastases in patients with BC and stated a significant correlation for Ki67.

However, Ziaei et al. [35] demonstrated that hormone receptors showed no relation with distant metastasis, but it was significantly correlated with poor survival. In addition, Khandani et al. [36] showed that hormone receptor status (i.e., ER, PR, and HER2) had no significant association with metastasis. Although in our patients there were no significant correlation between ER and PR status with brain metastasis, HER2 and P53 were markedly more positive among patients with metastasis. In line with our study, Payandeh et al. [37] revealed a significant correlation between the expression of HER2 and P53 with metastasis among Iranian patients with BC.

Although brain metastases are still considered to be highly fatal, enormous efforts are gradually leading to the establishment of safer and more effective treatments [38]. Hence, a better understanding of population variations sheds light on the aspects of the clinical and biological status of our patients to employ more sensitive screening methods, prioritize high-risk patients, and consequently achieve improved outcomes.

## Conclusion

In conclusion, patients with younger age, IDC, and HER2-positive and p53-positive receptors are at an increased risk of developing cerebral metastases. Also, it seems that mastectomy could not able to fully protect against cerebral metastases. Efficient work-ups are suggested to be undertaken for non-specific associated symptoms.

## Conflict of Interest

The authors declare that the research was conducted without any commercial or financial relationships that could be construed as a potential conflict of interest. Also, one of the authors of the article (Ehsan Jangholi) is the deputy editor of the journal. Based on the journal policy, he was completely excluded from any review process of this article, as well as the final decision.
